# Experimental study on the effects of interface dip angle on deformation failure of combined limestone–coal specimens

**DOI:** 10.1038/s41598-023-46186-w

**Published:** 2023-11-03

**Authors:** Faxin Li, Yisong Ding, Zhen Zhang, Zhiwei Zhang, Zhaojun Xuan, Qifeng Song

**Affiliations:** 1 Engineering Laboratory of Deep Mine Rockburst Disaster Assessment, Jinan, 250104 China; 2 Department of Civil Engineering, Graduate School of Engineering, Kyushu University, Fukuoka, 819-0395 Japan; 3 College of Energy and Mining Engineering, Shandong University of Science and Technology, Qingdao, 266590 China; 4 Shandong Kangge Energy Technology Co., Ltd., Jinan, 250098 China; 5 Shanxi Huoer Xinhe Coal Industry Co., Ltd., Changzhi, 046600 China; 6 Changle Planning and Compilation Research Center, Changle, 262400 China

**Keywords:** Civil engineering, Natural hazards

## Abstract

Uniaxial compression experiments of limestone–coal specimens at different inclination angles (0, 15, 30, 45, and 60°) were conducted using acoustic emission and three-dimensional, extension test digital image correlation, and full-field strain measurement systems to examine how dip angles affect deformation failure. The findings indicate that: (1) specimen groups demonstrate plastic yield characteristics in the pre-peak stage. However, slight variations exist due to inclination angles. (2) The localization zone for deformation evolution closely correlates to primary crack initiation and propagation within coal specimens and to slipping at the rock’s and coal’s interface. Failure in the coal specimen triggers rebound deformation in limestone when the rock coal inclination angle is set at 15°. Both the rebound deformation amount and its rate exhibit upward trends as a function of the inclination angle. (3) The percentage of pre-peak elastic property density in the combined specimen is augmented from 98.56 to 88.08% as the inclination angle augments and reduces to 75.80%. External energy’s conversion into missile performance shows an initial increase followed by a decrease. (4) The energy rate of the acoustic emission (AE) signal exhibits distinct temporal characteristics in the combined specimen that can be associated with quiet, active, and sudden increases.

## Introduction

China will maintain its dominant position in the future by relying primarily on coal as its primary energy source^1^. However, shallow reserves have been depleted owing to the rising demand for coal resources driven by social development, which has caused a shift toward the use of deep-mining techniques^2,3^. Unfortunately, a rise in unpredictable calamities, like sudden gas emissions and rock explosions, has been documented; this rise poses significant threats to property and human life. Research indicates that these disasters are often caused by overall instability in the combined structure of rock–coal seams resulting from mining activities^4–6^. Thus, understanding the deformation failure characteristics of combined structures is essential to prevent and control deep-mining disasters.

Currently, research on the combined structures of rock–coal seams has primarily focused on their simplification as combined specimens to conduct relevant studies on the mechanics and numerical simulations of rocks. Chen and Yin et al.^7–10^ performed uniaxial compression experiments on various characteristics of rocks, loading rates, and rock–coal height ratios, to investigate mechanical characteristics as well as the progressive failure mechanism of these combined specimens. Chai et al.^11^ conducted uniaxial and cyclic loading and unloading tests on composite samples with different roof lithologies, and studied the damage and failure process of composite samples under mining stress conditions, especially the mechanical response and damage evolution of coal and rock under cyclic disturbance conditions. Zhao et al.^12^ established an equivalent homogeneous model and stress state expression for coal–rock composites based on the principle of energy equivalence, proposed a general compressive shear failure criterion considering the bonding strength between coal and soft-rock interface, and revealed the effects of interface bonding strength, rock-sample thickness, and stress level on the failure behavior of these composites. Liu et al.^13^ proposed and verified a test method to obtain the stress and strain of middling coal samples in coal–rock combination samples by pasting strain gauges, established the damage constitutive models of coal samples in rock coal and rock–coal–rock combinations, and revealed the influence of rock samples on the mechanical behavior of coal samples. Zuo et al.^14^ conducted uniaxial and triaxial compression tests on rock–coal–rock composites containing weak coal samples, and studied their basic mechanical properties. Liu et al.^15^ conducted uniaxial compression tests on coal–rock combinations with different rock-sample strengths to investigate the influence and mechanism of rock-sample strength on the mechanical behavior and fracture mode of the combination. Huang et al.^16^ conducted cyclic loading and unloading tests on rock–coal–rock combinations under different loading rates to study the effects of loading rates on their stress–strain characteristics.

Yin et al.^17–19^ developed a mechanical model to study the uniaxial compression behavior of coal samples containing single-joint rock–coal composites in numerical simulation research. Furthermore, they utilized the numerical software PFC^2D^ to simulate uniaxial compression tests about these coal samples with single joint composites and investigated how the joints’ presence affected the strength, AE patterns, and failure characteristics. Li et al.^20^ used PFC^2D^ particle-flow numerical software to study the influence of different forms of parallel precracks on the mechanical properties of composite coal and rock, and revealed the influence of parallel precracks on the mechanical properties of coal and rock composites. Guo et al.^21^ used PFC^2D^ particle-flow software to simulate uniaxial compression tests of rock–coal–rock composite specimens at different coal thicknesses, and revealed the influence of coal thickness on the failure mechanical behavior of rock–coal–rock composite specimens. Zhao et al.^22^ used PFC^2D^ particle-flow software to simulate uniaxial and biaxial compression tests of rock–coal composite specimens under different rock–coal dip angles, thus revealing the influences of the interfacial dip angle and fractal characteristics on the mechanical and failure characteristics of these specimens. Nie and Zhao et al.^23,24^ used PFC^2D^ particle-flow software to simulate uniaxial compression tests of rock–coal composite specimens at different rock–coal height ratios and dip angles, and revealed the influence of rock–coal height ratios and interfacial dip angles on the mechanical and failure characteristics of composite specimens. Zhao et al.^25^ conducted simulations using the numerical software FLAC^3D^ to investigate the strength and failure characteristics of rock–coal–rock specimens subjected to triaxial and uniaxial compression experiments at various strength ratios.

The aforementioned research studies are of utmost importance in comprehending coal–rock seam-structural failure and strength properties. However, varying geological conditions can influence the deformation failure characteristics based on changes in angles of inclination at interfaces between rocks and coal. Hence, it should investigate how the inclination angle impacts deformation failure. Consequently, this study focuses on the investigation of five sets of combined limestone–coal specimens at different rock–coal inclination angles (0, 15, 30, 45, and 60°). Uniaxial compression tests were conducted on these specimens using AE and three-dimensional (3D), XTDIC, full-field strain measurement systems. Underlying mechanisms uncovering how deformation failure is affected by interfacial inclination angles at various rock-coal configurations are provided by comparing factors such as energy evolution, AE patterns, deformation failure behavior, and strength among these specimens at various inclination angles.

## Preparation and testing scheme for specimens

### Specimen preparation

**Figure 1:**
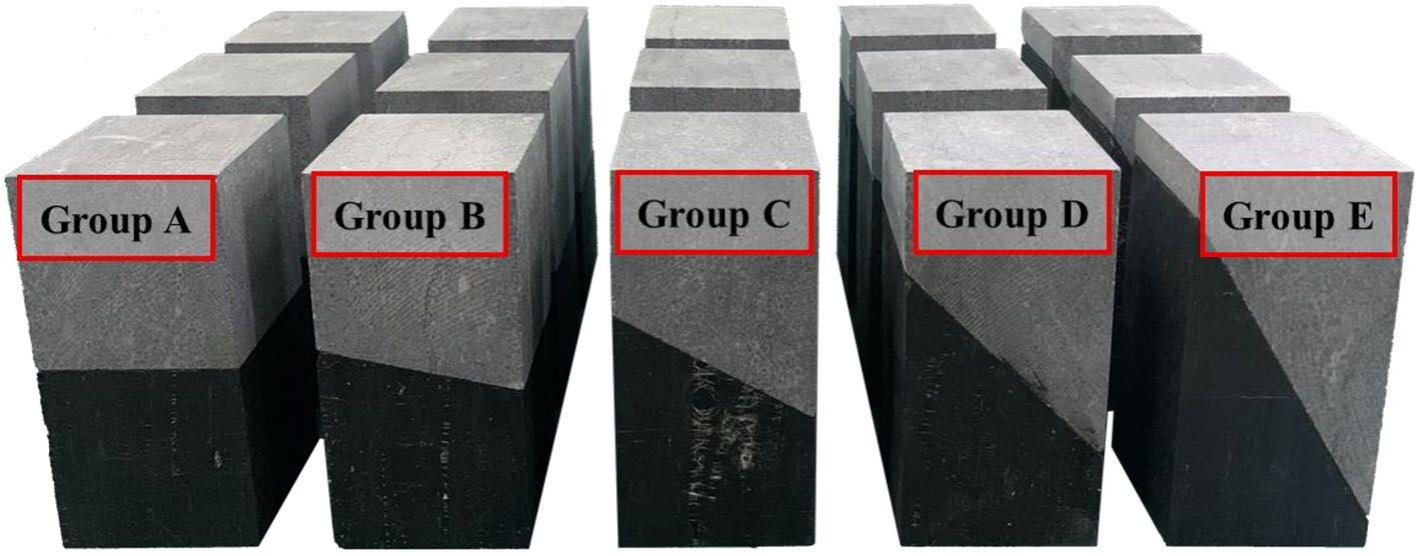
Combined limestone–coal specimens.

Tangshan Coal Mine offered coal, limestone, and other specimens utilized in this experiment. All limestone and coal specimens were extracted from identical blocks to minimize the dispersion’s impact on the test outcomes. An SCQ-A was employed to automate fully the rock cutter (frequency-conversion type) and cut limestone and coal into 50 × 50 mm specimens following the relevant procedure executed to determine the physical and mechanical properties in a range of inclination angles (0–60°) at regular 15° intervals. Subsequently, each specimen’s end face was ground using an SCM200 double-end grinding machine (common type) until it achieved a smooth surface. The nonparallelism between corresponding end faces did not exceed 0.05 mm, while the axial deviation remained within 0.25°. Finally, epoxy resin AB glue was used to bond the limestone and coal specimens together into square cylinder specimens (with sizes of 50 mm × 50 mm × 100 mm) following established literature standards. These specimens were then classified into sets A, B, C, D, and E, as depicted in Fig. 1.

### Test scheme

**Figure 2:**
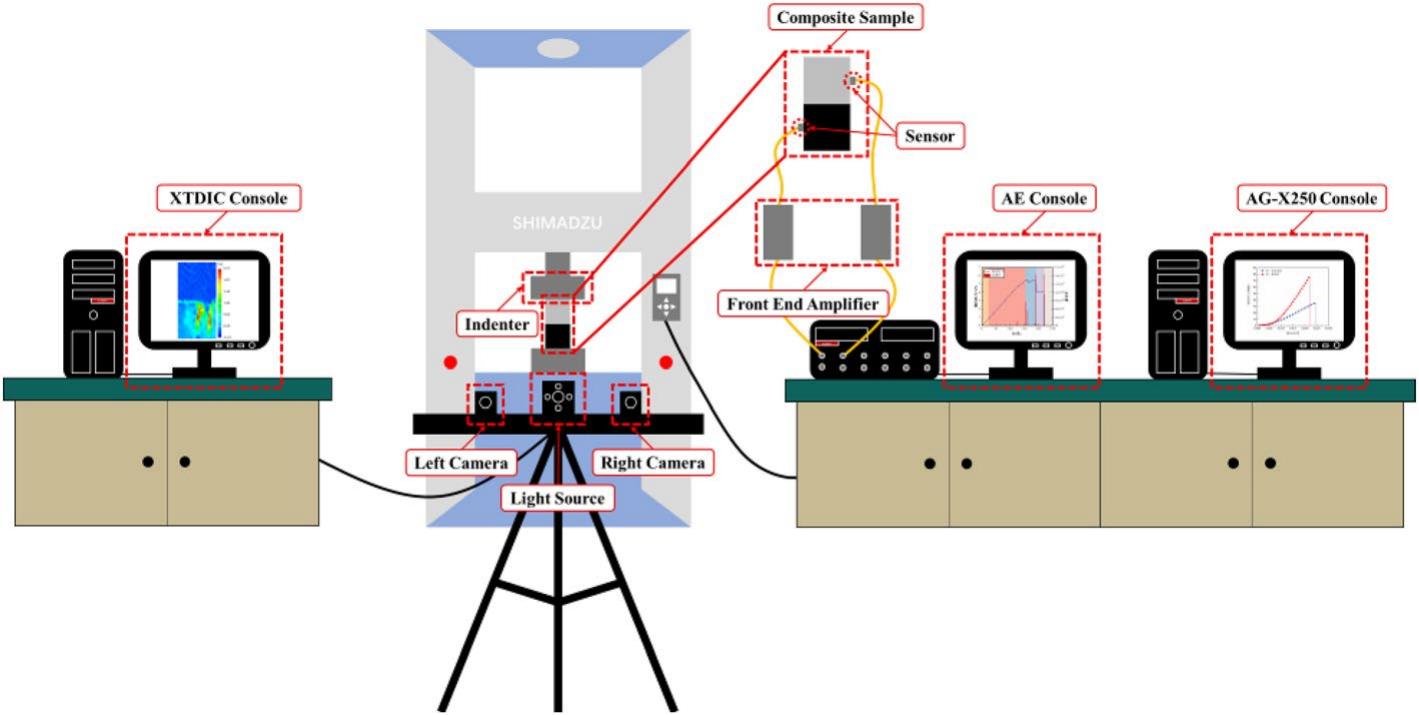
Testing equipment and system setup.

The experimental setup, as depicted in Fig. 2, consists of an electronic universal testing machine manufactured by Shimadzu with the model number AG-X250, a PCI-2 AE system from the MISTRAS series, and XTDIC systems for measuring 3D strains with full-field coverage. During the experiment, we conducted loading, AE monitoring, and strain measurement simultaneously to confirm synchronized data processing and analysis across all three systems^26,27^.

An electronic universal testing machine (Shimadzu AG-X250 model), was utilized to perform uniaxial compression tests on combined specimens at various rock–coal inclination angles. A method for controlling loading through displacement was employed after specimens reached their breaking point throughout the experiment. The loading rate was 0.005 mm/s, with data recorded every 10 ms and a sensitivity of 1%. This implies that the applied stress decreased to only 1% of its maximum value before the test was automatically terminated.

Additionally, a 3D, full-field strain measurement system called XTDIC was employed to monitor the combined specimens’ deformation characteristics under uniaxial compression. This noncontact optical system allowed the assessment and examination of surface morphology, movements, and strain in objects. The measured results are directly displayed. Speckle field’s manual application was performed by evenly spraying white matt paint onto the specimen’s surface before conducting the test. Subsequently, black matt paint was randomly sprayed on top to create black speckles. Consequently, a black speckle field formed against the combined specimen’s white background^28,29^. A charge-coupled device camera with 5 million pixels captured strain diagrams at an acquisition frequency of 4 frames per second during testing.

The PCI-2 AE system from the MISTRAS series was utilized to monitor the combined specimens’ AE properties during uniaxial compression. The R3α sensor model with a resonant frequency range of 20–100 kHz was selected using the main amplifier of 40 dB. A threshold level of 45 dB along with a floating threshold of 6 dB was set, with sampling frequencies set at 10^6^ times per second. The limestone surface had an AE sensor securely attached using tape to ensure an optimal reception signal by the sensor^30^. Vaseline was applied between them to minimize any differences in acoustic impedance and energy loss at their interface. A lead-breaking coupling test confirmed that the amplitude signal received by the sensor exceeded 90 dB^30^ before the test was conducted.

### Strength characteristics of limestone–coal composite

**Figure 3:**
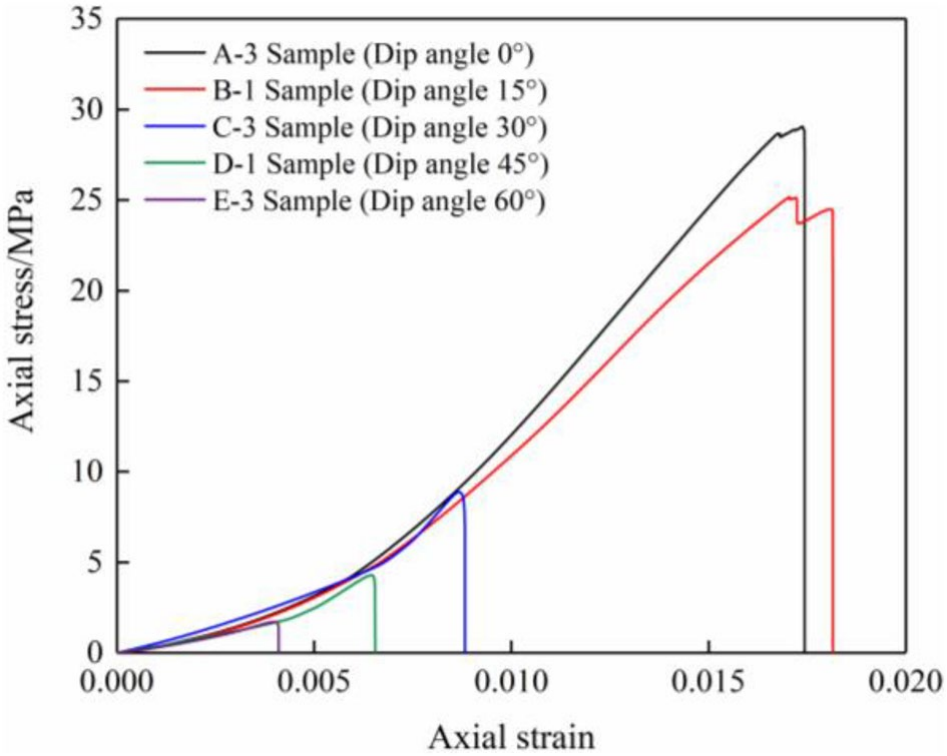
Uniaxial compressive stress–strain curves of limestone–coal specimens at various inclination angles.

Figure 3 displays the stress–strain curves of the combined specimens (comprising limestone and coal) which underwent uniaxial compression at various inclination angles.

The stress–strain curves of the combined limestone–coal specimens at various inclination angles were observed to undergo commencing consolidation, linear flexibility, plastic deformation threshold, and post-peak collapse. The pre-peak stage showed plastic yield characteristics in all specimen groups but exhibited slight differences owing to the inclination angle’s influence. A small “stress drop” phenomenon was observed during the plastic yielding stage when the inclination angle was ≤ 15° and a smooth trend was noted at higher inclination angles (≥ 30°). Split ejection failure primarily occurred at 15° after the peak; this resulted in small-scale ejection failures during the plastic yield process. However, slip failure along the interface became more evident after the peak at higher inclination angles (≥ 30°) and resulted in a change from splitting catapult to sliding failure as the mixed specimen’s inclination angle increased.

**Figure 4:**
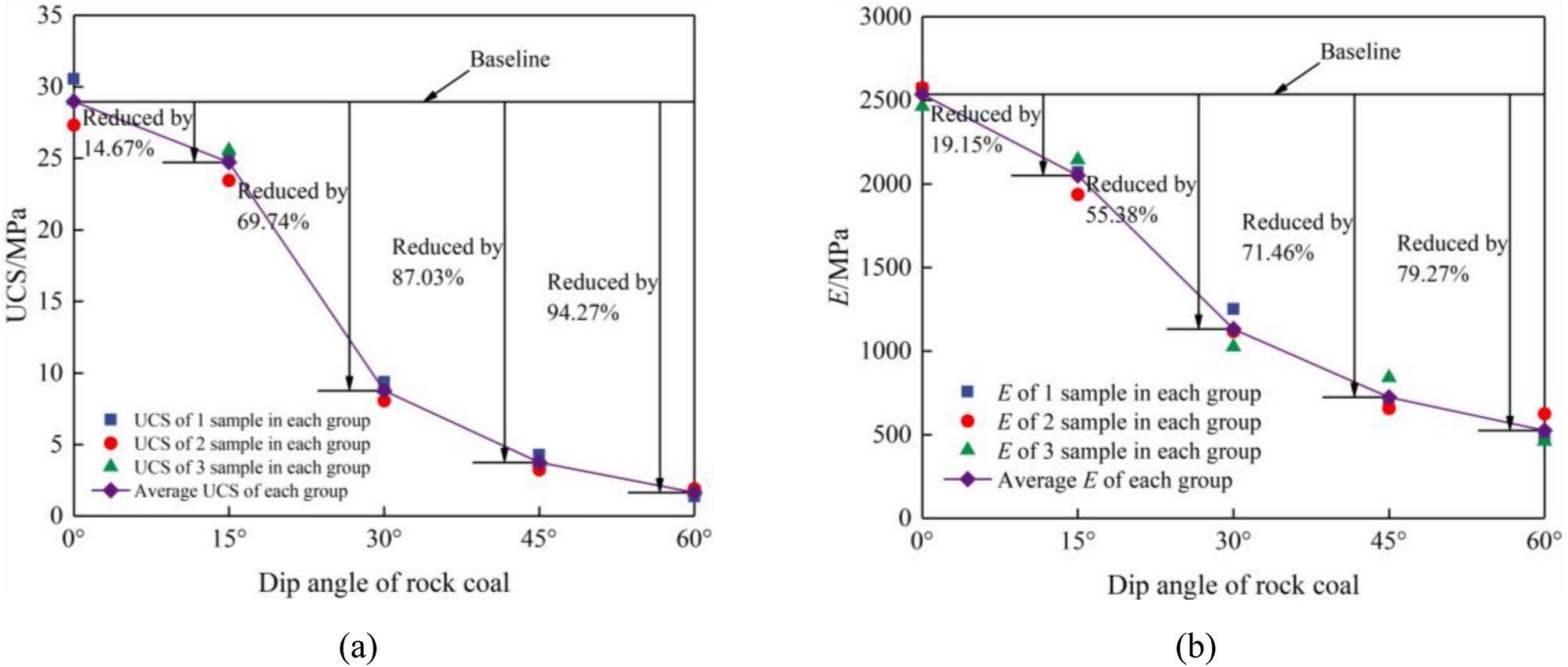
Comparison of UCS and* E* of combined limestone–coal specimens at different inclination angles. (**a**) UCS and (**b**)* E* comparisons.

Table 1 lists uniaxial compression test results of limestone–coal combined specimens with different inclination angles. A comparison between uniaxial compressive strength (UCS) and elastic modulus (*E*) values is derived at different inclination angles (Fig. 4a,b).

**Table 1:** Uniaxial compression test results of combined limestone–coal specimens at different inclination angles.

Set	Size/mm	UCS/MPa	*E*/MPa
Set A	50 × 50 × 100	27.33–30.54 Average = 28.98	2464.07–2574.16 Average = 2536.45
Set B		23.46–25.56 Average = 24.73	1936.70–2144.96 Average = 2050.74
Set C		8.07–9.36 Average = 8.77	1025.94–1251.38 Average = 1131.78
Set D		3.24–4.28 Average = 3.76	657.19–841.39 Average = 723.90
Set E		1.36–1.91 Average = 1.66	460.98–625.37 Average = 525.89

When comparing limestone–coal combined specimens at the inclination angle of 0° to those at 15, 30, 45, and 60°, respective reductions in UCS are observed by approximately 14.67%, 69.74%, 87.03%, and 94.27% (Table 1 and Fig. 4). Similarly,* E* decreased by approximately 19.15%, 55.38%, 71.46%, and 79.27%. Henceforth, an increase in the rock–coal inclination angle resulted in a reduction of both *UCS* and *E* for the combined specimen. The observed inclination angles ranged from 15° to 30°, where an instant change occurred in the decline of UCS and *E*. This phenomenon could be attributed to the amplified rock–coal inclination angle. The combined specimen’s primary mode of failure occurred along the interface due to the influence of the interfacial inclination angle effect. Various analytical methods were employed to investigate further how interfacial inclination angles affected deformation failure within the combined specimen.

The conditional formula (1) for the failure of combined rock–coal specimens as a function of the inclination angle subjected to confining pressures has been proposed in the literature^31^. As only the uniaxial compression test was conducted in this study, formula (2) can be obtained based on formula (1) for the combined specimen’s failure conditions.

1 $$\sigma_{1} = \sigma_{3} + \frac{{2\left( {c_{\omega } + \sigma_{3} \tan \varphi_{\omega } } \right)}}{{\left( {1 - \tan \varphi_{\omega } \cot \beta } \right)\sin \left( {2\beta } \right)}}$$

2 $$\sigma_{1} = \frac{{2c_{\omega } }}{{\left( {1 - \tan \varphi_{\omega } \cot \beta } \right)\sin \left( {2\beta } \right)}}$$

where $\varphi_{\omega }$ represents the rock–coal interface’s internal friction angle, $c_{\omega }$ represents the rock–coal interface’s cohesion, $\beta$ is the angle between the horizontal plane and rock–coal interface, $\sigma_{3}$ represents the combined specimen’s minimum principal stress, and $\sigma_{1}$ represents the combined specimen’s maximum principal stress.

The cohesion and internal friction angles remained constant due to the utilization of limestone and coal specimens in the experiment. These assumptions were solely employed to describe the combined specimen’s overall characteristics. The findings indicated an inverse relationship between the rock and coal’s inclination angle and the combined specimen’s uniaxial compression strength. Rising inclination angles reduced the failure strength for these combined specimens under uniaxial compression conditions. Furthermore, it was observed that variations in interface inclination angle reduced the strength of the combined samples.

### Deformation failure characteristics of limestone–coal composite

#### Evolution properties of the deformation field

**Figure 5:**
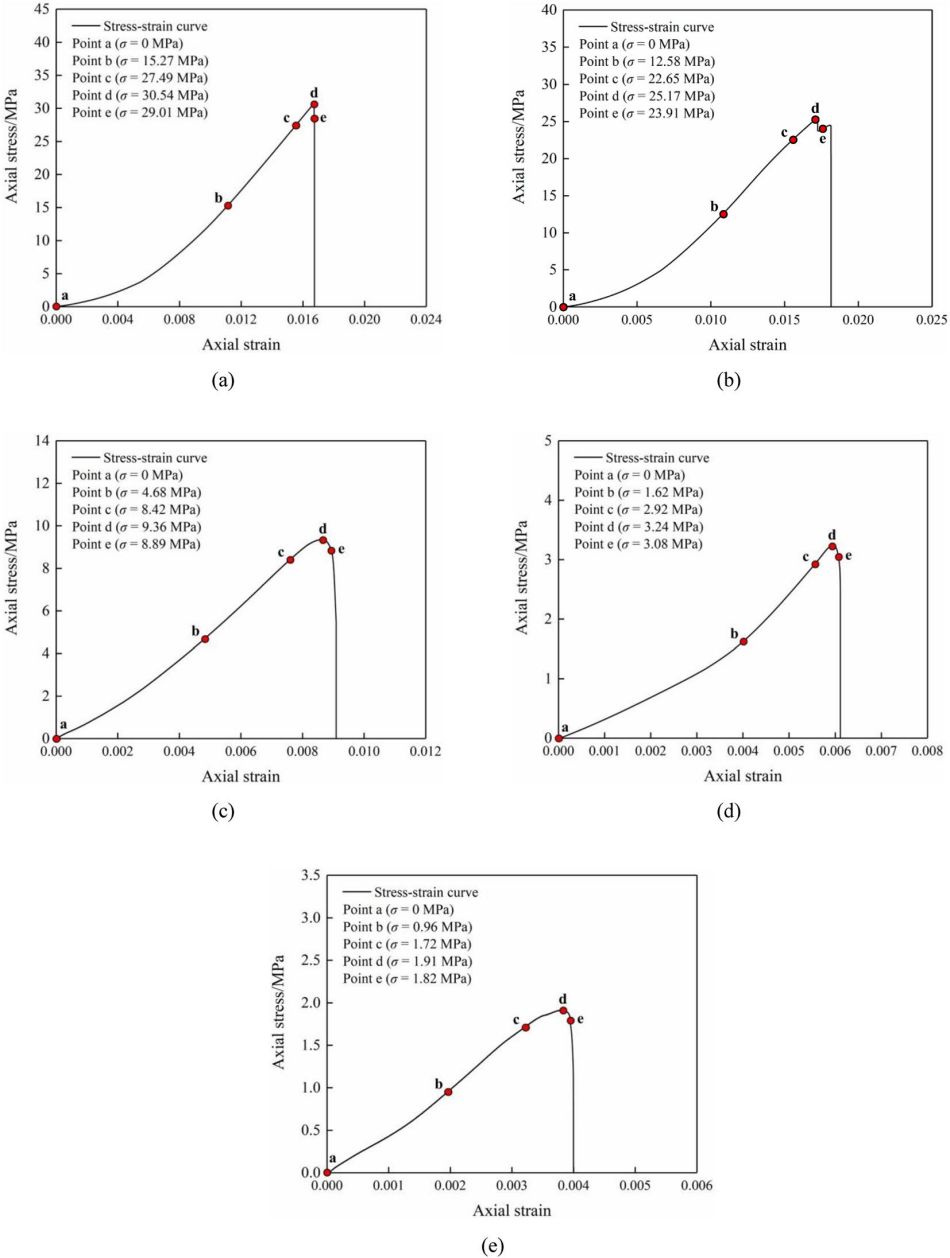
Selection of characteristic points of limestone–coal combined specimens at different inclination angles. Combined specimens (**a**) A-1, (**b**) B-1, (**c**) C-1, (**d**) D-2, and (**e**) E-2.

**Figure 6:**
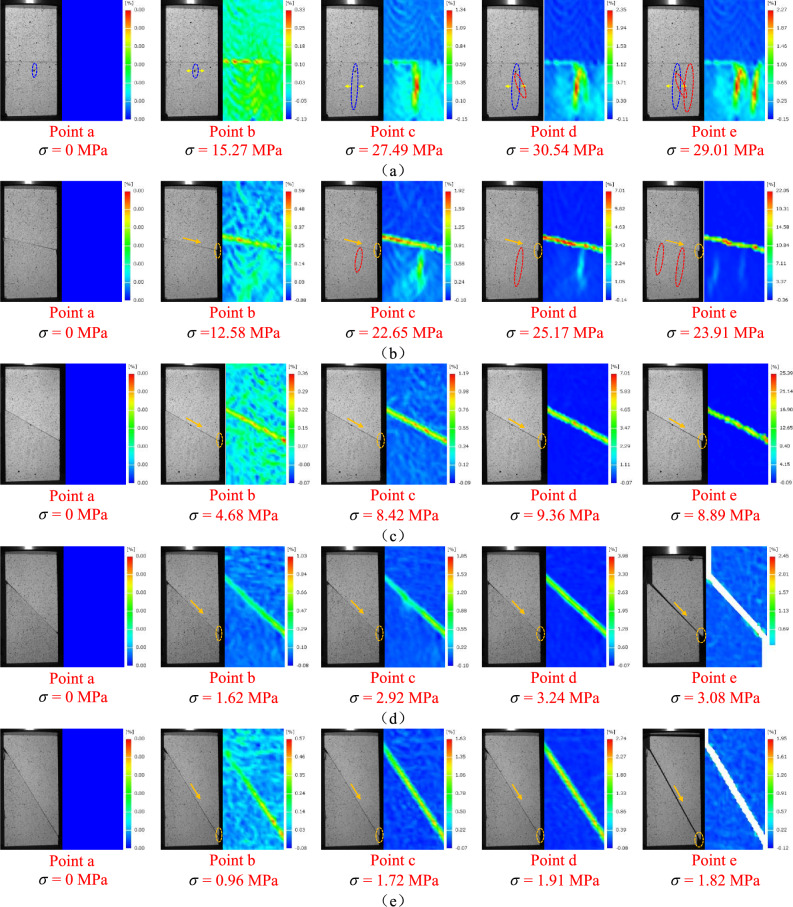
Evolution cloud diagram of the maximum principal strain field of combined limestone–coal specimens at different inclination angles. Combined specimens (**a**) A-1, (**b**) B-1, (**c**) C-1, (**d**) D-2, and (**e**) E-2.

During the uniaxial compression test of the combined limestone–coal specimens at various dip angles, consistent results were obtained for three specimens in each group. As a result, one specimen was chosen to analyze the deformation field’s evolution law. The cloud diagram shows how characteristic points were chosen and how the maximum principal strain field evolved for these specimens (Figs. 5 and 6). Five characteristic points were identified on each stress–strain curve to analyze the combined specimen’s deformation failure characteristics: points a-e represent the axial stress (0 MPa), 50 and 90% of peak stress (before reaching the peak), peak stress, and 95% of the peak stress (after reaching the peak), respectively.

The digital speckle correlation technique was employed to identify point a as the corresponding feature point of the reference image. Additionally, points b–e were recognized as the deformed image’s corresponding feature points using the same method. The blue ellipses represent pre-existing cracks, red ellipses represent newly formed cracks or the extension of pre-existing cracks leading to the creation of new cracks, yellow arrows indicate crack propagation, orange arrows, and ellipses represent interfacial sliding, and $\sigma$ denotes the axial stress associated with each characteristic point. Characteristic point b was observed during the initial phase of the stress–strain curve (in the linear elastic region). No significant development of the original cracks occurred at this stage. The strain distribution within the combined specimen appeared to be relatively homogeneous, and only a localized deformation band formed at the interface. Characteristic point c marked the primary crack formation’s initiation in the cases of the combined specimens A-1 and B-1 as the axial stress gradually increased. Furthermore, deformation localization bands emerged in their respective regions, primarily within the coal specimen. However, more extensive development of deformation localization zones was observed at the interfaces of the combined specimens C-1, D-2, and E-2. The values of $\sigma$ for the combinations A-1, B-1, C-1, D-2, and E-2 were 27.49 MPa, 22.65 MPa, 8.42 MPa, 2.92 MPa, and 1.72 MPa, respectively. The maximum principal strains were found to be 0.0134 (A-1), 0.0192 (B-1), 0.0119 (C-1), 0.0185 (D-2), and 0.0163 (E-2). Characteristic point d was identified as the stress–strain curve’s peak point, where primary cracks in the combined specimens A-1 and B-1 continuously expanded, intersected, and generated new cracks. Slip further developed at the interface of the combined specimens C-1, D-2, and E-2. Additionally, a nonuniform distribution of the maximum principal strain field existed and an increase in its value was documented. The values of $\sigma$ for the composites A-1 and B-1 were 30.54 MPa and 25.17, respectively, with corresponding maximum principal strains of 0.0235 and 0.0701. For the combination specimens C-1, D-2, and E-2, the stress values were 9.36, 3.24, and 1.91 MPa, respectively, and the maximum principal strains were 0.0701, 0.0398, and 0.0274, respectively.

The maximum principal strain values of the combined specimens A-l, B-l, C-l, D-2, and E-2 increased by up to 75%, 265%, 489%, 115%, and 68% at point d compared with those at point c. Furthermore, the rate of the maximum principal strain value first increased and then decreased at increasing inclination angles at the rock–coal formation interface. In addition, the combined specimens A-1 and B-1 experienced axial stress, which caused the primary crack tip in the coal specimen to initiate cracking and expansion. This resulted in the formation of a macro tensile crack and an increase in the deformation localization band’s length and width. Point e on the stress–strain curve was identified to be within the post-peak failure stage. Furthermore, connections were observed between the original and new cracks in the combined coal specimens A-1 and B-1. As a result, elongation, intersection, and connection of deformation localization bands occurred and led to failure. Conversely, interfacial slip accompanied by deformation localization bands’ violent evolution was observed for the combined specimens C-1, D-2, and E-2, and resulted in interfacial slip failure. The corresponding $\sigma$ values for the combined specimens A-1, B-1, C-1, D-2, and E-2 were found to be 29.01, 23.91, 8.89, 3.08, and 1.82 MPa, respectively, while the maximum principal strain values were equal to 0.0227, 0.2205, 0.2539, 0.0245, and 0.0195, respectively.

In summary, the deformation localization zone’s formation and expansion in combined specimens were closely influenced by the primary cracks’ initiation and propagation within coal specimens as well as movements along rock–coal interfaces.

#### Examination of the development patterns in the localized deformation zone’s displacement evolution

**Figure 7:**
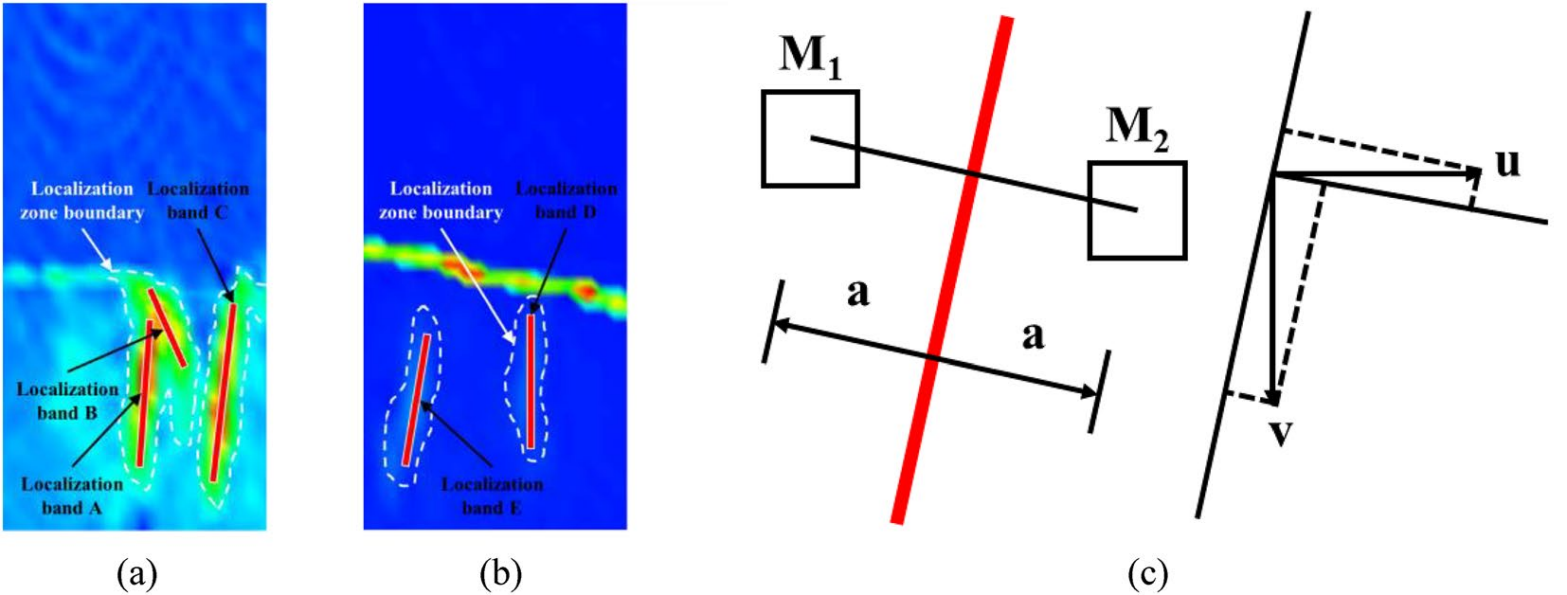
Summary of the method used for analyzing the movement evolution in bands for localizing deformation in combined specimens and relevant results. Combined specimens (**a**) A-1, (**b**) B-1, and (**c**) schematic of the method used to analyze staggered displacements surrounding the zone of localized deformation on either side.

**Figure 8:**
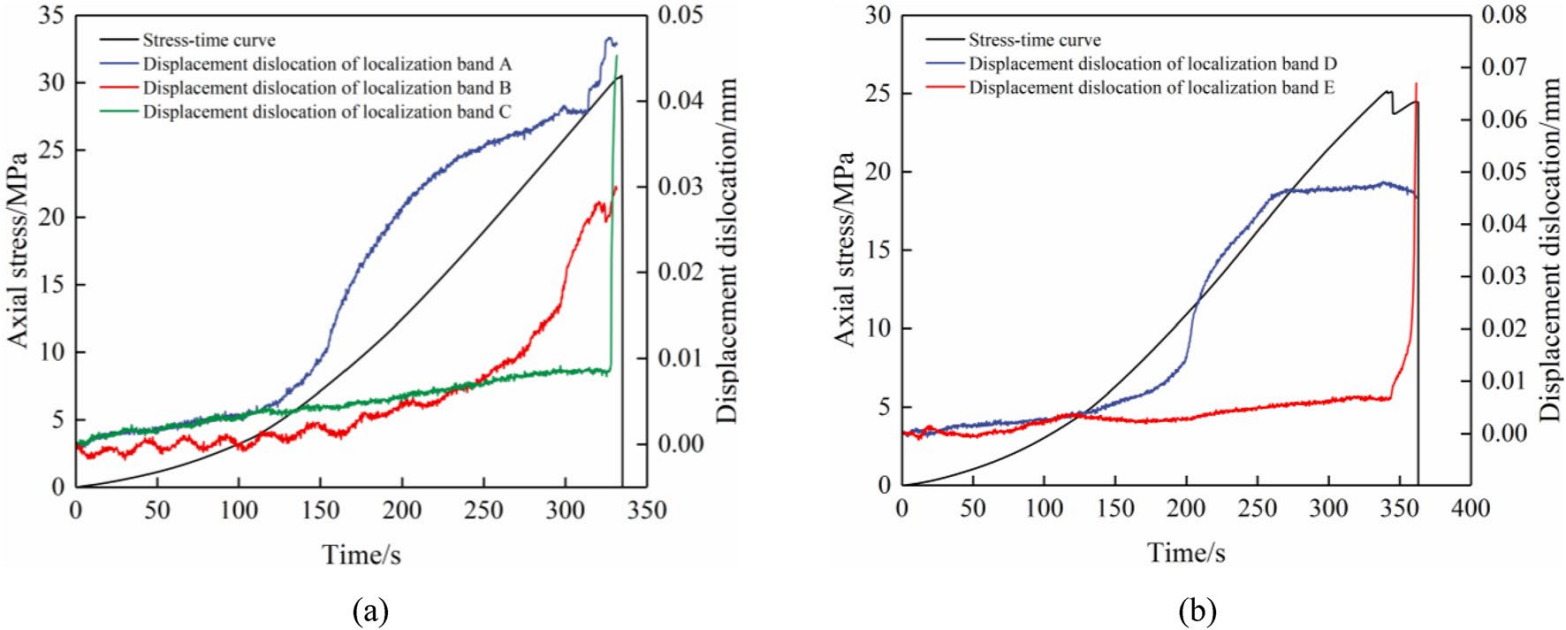
Evolution curves depicting movement dislocations of zones of localized deformation in combined specimens. Combined specimens (**a**) A-1 and (**b**) B-1.

The displacement evolution analysis method was employed to study the localization zone’s characteristics by quantitatively analyzing the deformation field progression in a combined specimen that comprised limestone and coal. The deformation localization band (only at the crack) in the cloud diagram of the maximum principal strain was identified as the combined specimen’s final failure (as depicted in Fig. 7a,b) by referring to its definition^32^. Utilizing the movement dislocation method proposed by Song et al.^33^ on the band for localizing deformations on both sides, with the distance (denoted by “*a*”) between the two sides of its identification line being equal to 2 mm, we determined *M*_1_ and *M*_2_ as center points for selected pixel points. The variables *u* and *v* represented their respective movement components (see Fig. 7c). Consequently, the displacement was calculated within this deformation localization band for the combined specimen, with results presented in Fig. 8. Note that positive values indicate a counterclockwise direction for displacements.

Figure 8 shows the movement dislocation progression in the localizing deformation zones of the combined limestone–coal specimens; these can be categorized into three phases: microalteration, gradual enlargement, and rapid expansion. These stages are primarily influenced by the establishment, extension, and connection of the deformation localization zone. Initially, no deformation localization band was observed in the combined specimen, and displacements A-E within these bands were almost negligible (approximately 0 mm), which indicates a phase of microalteration. As the axial stress increased, the formation of zones of localized deformation occurred first in the coal specimen’s main crack areas before they propagated and enlarged along the direction aligned with maximum principal stress. However, owing to forming time variations and development and expansion processes associated with the localized distortion region, the initiation times of different stages differed accordingly.

Among them, the combined specimen A-1 exhibited negligible displacement (0 mm) in the deformation localization band before it was subjected to a load for 118 s. Subsequently, the displacement gradually increased. Following 154 s of loading, there was an acceleration in the displacement dislocation of deformation localization band A; however, its growth rate gradually decreased. Deformation localization bands B and C continued to experience slow growth at this stage. An instantaneous increase existed in the displacement within the deformation localization band B upon reaching 295 s of loading. Nevertheless, the combined specimen did not enter the post-peak stress drop stage at this point. It was only when the load reached 328 s that an instant increase existed in displacement dislocation within deformation localization band C; this finding indicates that it played a crucial role in the failure of the combined specimen A-1. Both deformation localization bands D and E initially exhibited negligible displacement (0 mm) before they were loaded for 116 s; the combined specimen B-1 was then considered. A gradual increase in displacement dislocation within the deformation localization band D was documented until it instantly increased at the loading time of 200 s (after the end of the loading). However, no significant displacement dislocation change occurred and its value remained practically unchanged during this period while deformation localization band E continued to experience slow growth up to the loading time of 270 s. The internal structure underwent adjustments to accommodate axial stress when the load reached its maximum stress point. A “stress increase” phase occurred during which the displacement dislocation of deformation localization band D remained relatively stable, although no immediate drop occurred in the stress–strain curve. However, an abrupt increase in the dislocation of deformation localization band E occurred at 344 s that indicates that the combined specimen entered a post-peak softening stage. This observation suggests that deformation localization band E played a crucial role in influencing the B-1 combined specimen’s failure.

The sudden increase in the movement dislocation of zones for localized deformation led to the deformation and combined specimen’s failure that resulted in a reduction in its bearing capacity and a corresponding decrease in axial stress. Hence, this abrupt increase in deformation localization bands can serve as a significant indicator for the prediction of the combined specimens’ failure and deformation. Furthermore, the maximum displacement dislocation was found to be influenced by the positions and directions of these bands. Generally, these bands exhibited relatively large maximum displacement dislocations when located at either the ends or sides of the specimens. For instance, band A yielded a maximum displacement dislocation of 0.047 mm, while bands C and E yielded displacements of 0.045 mm and 0.067 mm, respectively. Conversely, these bands displayed relatively smaller maximum displacement dislocations when positioned at the specimen’s middle section; for example, band B had a value of 0.030 mm whereas band D was equal to 0.048 mm.

#### Examination of the developed patterns during the evolution of displacement in the localized deformation zone

**Figure 9:**
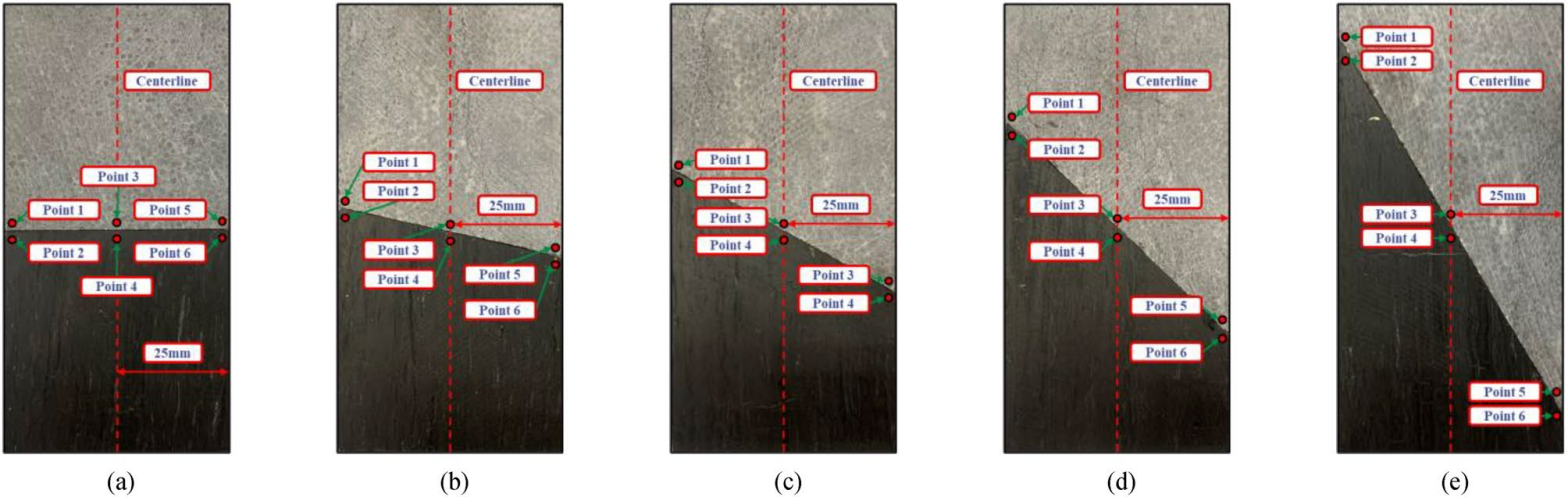
Arrangement of monitored locations to track displacement variations in limestone and coal samples within the interface of the combined specimen. Combined specimens (**a**) A-1, (**b**) B-1, (**c**) C-1, (**d**) D-2, and (**e**) E-2.

**Figure 10:**
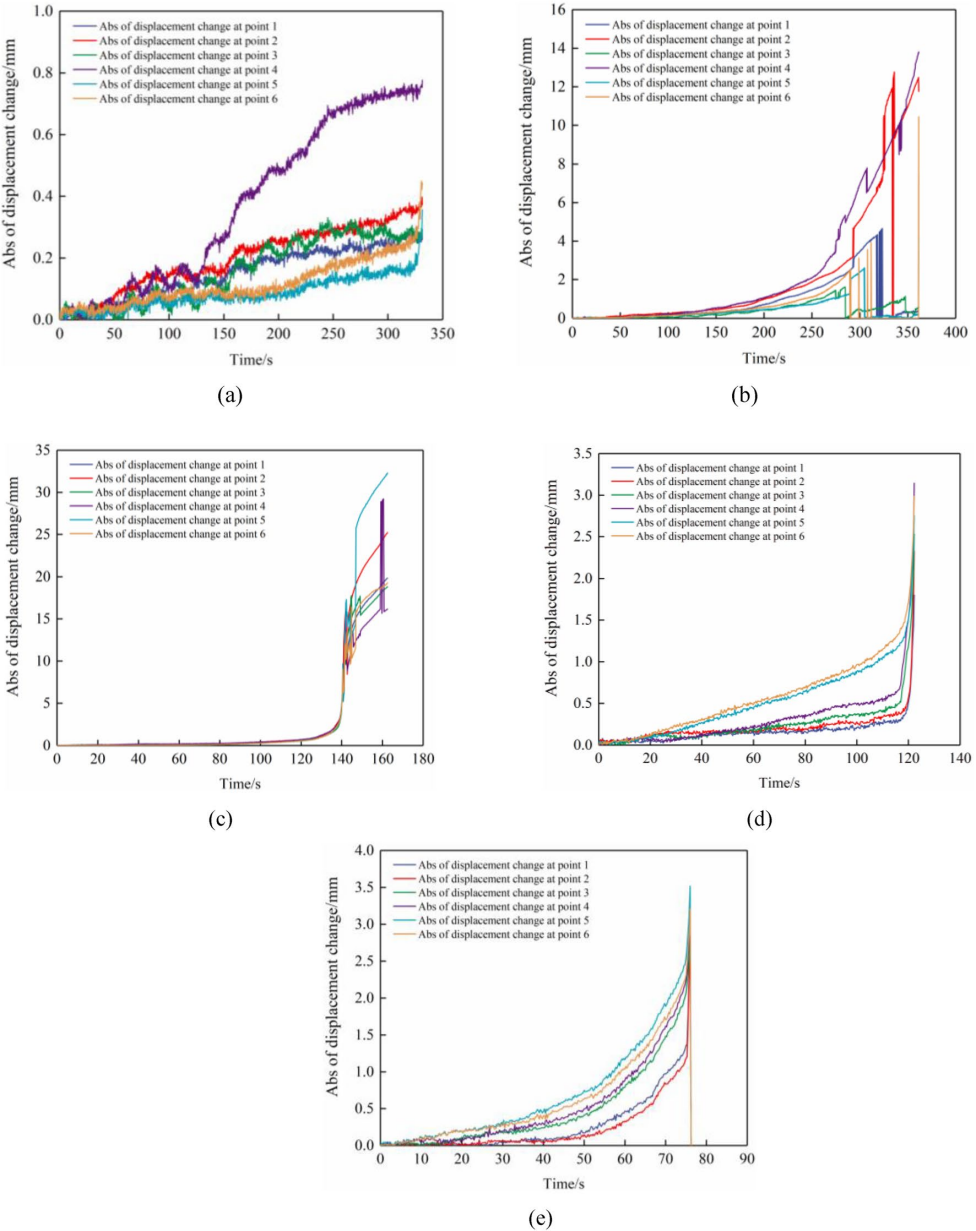
Plots depicting the changes in displacement of limestone and coal samples near the interface. Combined specimens (**a**) A-1, (**b**) B-1, (**c**) C-1, (**d**) D-2, and (**e**) E-2.

To assess the impact of the interface between the limestone and coal specimens on the deformation and failure of the combined specimens, we repositioned the monitored points at the interface, as depicted in Fig. 9. The absolute displacement change at monitored points 1–6 at the interface is illustrated in Fig. 10.

Specimens’ inconsistent deformation at the interface was attributed to the differing mechanical properties of the composite limestone–coal specimens. However, to maintain overall stability, stress was generated at the interface to control or facilitate the deformation of limestone–coal specimens. This phenomenon is referred to as the inclination effect at the interface. During testing, it was observed that while limestone had a higher elastic modulus compared with coal, its Poisson’s ratio was lower. As a result, tensile stress occurred at the limestone area’s junction, and thus promoted deformation. Conversely, compressive stress developed in the interface’s coal specimen area, thus limiting its deformation.

As depicted in Fig. 10, displacement exhibited a consistent, and gradual growth tendency at observation points 1, 2, 3, 4, 5, and 6. Notably, before loading, there was consistency in absolute movement changes at the observation points 1 and 2 and 5 and 6 for the combined specimen A-1. Following a load duration of approximately 130 s, an “abrupt increase” was observed in absolute movement changes at observation point 4; however, growth rates slowed down by 250 s. The disparity in absolute movement changes between observation points 3 and 4 primarily resulted from the expansion, transfixion, and dislocation of the deformation localization bands within the combined specimens. Particularly noteworthy is the deformation localization band A within coal specimens. In the case of combined specimen B-1, the evolution of absolute movement changes was consistent among all six monitored points up to 260 s (before loading commenced). However, outcomes fluctuated significantly at the loading time of 260 s, thus resulting in instances such as either both “sudden rising” or “sudden decreasing.”

In addition, absolute movement changes at monitoring points 1, 2, 3, 4, 5, and 6 differed between combined specimens C-1, D-2, and E-2 compared with combined specimens A-1 and B-1. When rock–coal inclination angles exceeded or became equal to 30°, interfacial slip failure was primarily observed in combined specimens. As a result, the absolute movement changes were consistent at monitored points 1–6 during loading. The “sudden change” in absolute movements occurred only at the moment when combined-specimen destruction took place. Moreover, an increase in rock coal inclination angle led to a decrease in time for this “sudden change” from 140 to 75 s.

In summary, limestone–coal collaborative deformation was influenced by interfacial inclination angles. When the rock–coal inclination angles were ≤ 15°, specimens underwent initial destruction under axial stress due to combined specimens’ relatively low strength. This led to the rapid development of the specimen’s internal deformation localization zone, expansion, and connection, thereby promoting the interface’s deformation. Moreover, noncooperative deformation occurs at limestone–coal specimen interfaces when the inclination angles are ≥ 30° as a result of slip failure.

#### Exploring the rebound deformation characteristics exhibited by limestone

**Figure 11:**
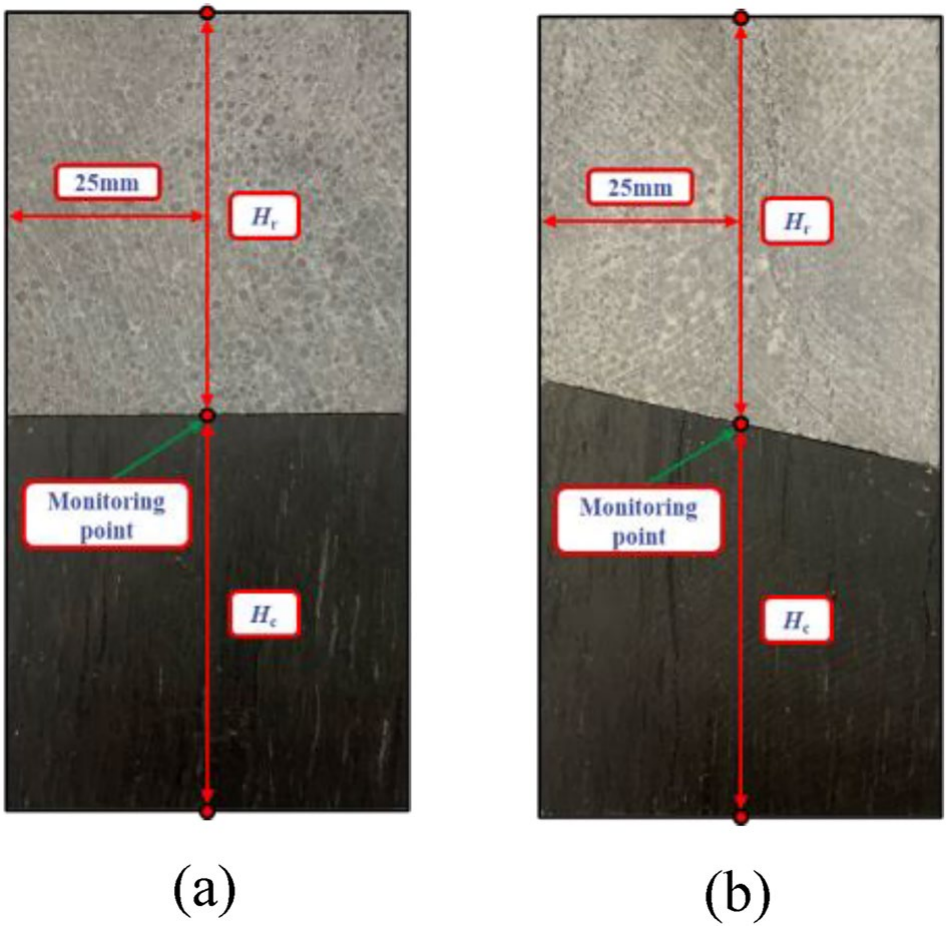
Layout of monitoring points for limestone–coal composite samples at different dip angles. Combined specimens (**a**) A-3 and (**b**) B-2.

The mechanical properties of coal–rock specimens differed from each other. When subjected to axial stress, partial specimens experienced fracturing (fracture body), while other parts underwent rebound deformation (rebound body). In this experiment, it was observed that the strength of coal specimens was significantly lower compared with that of the limestone specimens. As combined specimens approached their strength limit, fracture occurred in coal specimens while limestone remained in an elastic state. Coal fractures resulted in limestone’s rebound deformation. Therefore, we can classify limestone specimens as rebound bodies and coal specimens as fracture bodies^34^. However, for combined specimens with inclination angles equal to or exceeding 30°, sliding became more dominant along interfaces. Interestingly, no breakage occurred in coal specimens; there was no rebound deformation observed in limestone either. Henceforth, to investigate how inclination angles at interfaces affect coal and rocks at inclination angles less than or equal to 15°, monitoring points were strategically placed at the interfaces of coal and limestone specimens to observe any changes occurring in height *H*_r_ for limestone specimens as well as height *H*_c_ for coal specimens, as illustrated in Fig. 11.

**Figure 12:**
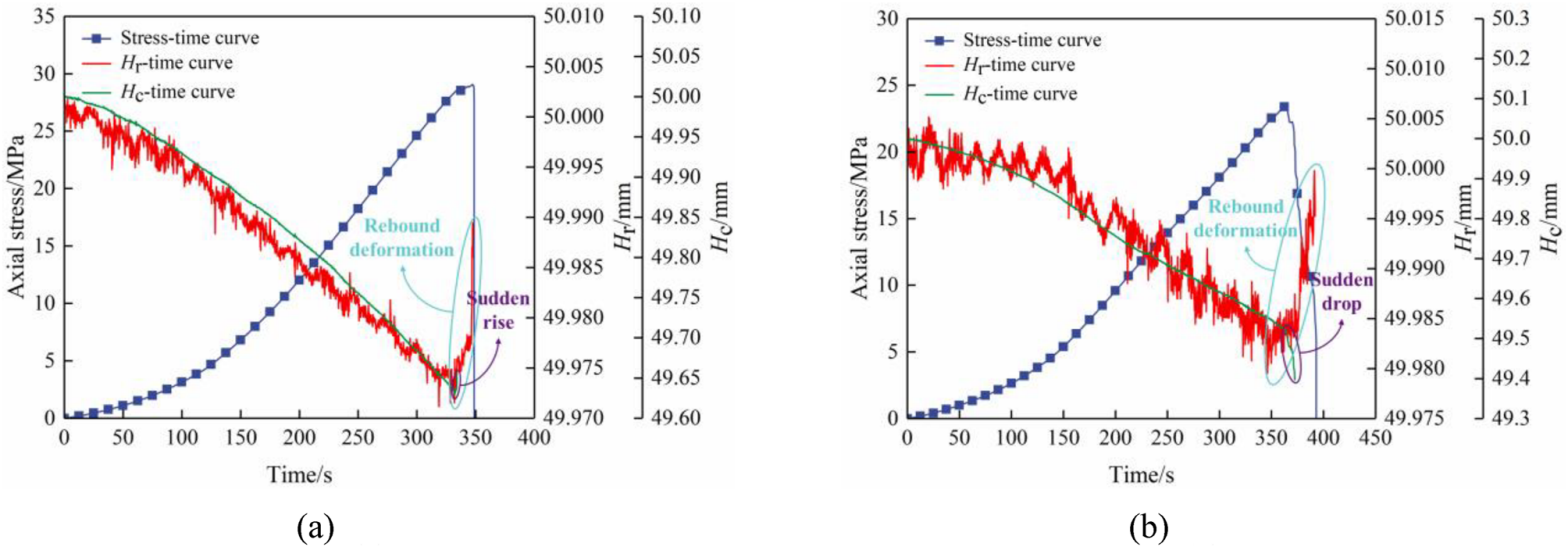
Time-dependent curves of axial stresses,* H*_r_, and *H*_c_ of limestone–coal composite samples at different dip angles. Combined specimens (**a**) A-3 and (**b**) B-2.

Figure 12 shows variations in axial stress, *H*_r_, and *H*_c_ over time. Both *H*_r_ and *H*_c_ exhibited a general downward trend with variations during limestone specimens’ failure process at various inclination angles. The primary factors influencing variations in composite samples’ *H*_r_ and *H*_c_ were primarily attributed to various characteristics, such as initial cracks’ length and quantity present within both coal and limestone specimens.

In the primary fractures of the coal specimens, there was a significant drop in the stress–strain curve peak for the combined specimens. Furthermore, *H*_r_ experienced varying degrees of increase while* H*_c_ exhibited a nonlinear decrease, thus indicating that the failure of coal specimens resulted in limestone’s rebound deformation. For instance, when combined specimen A-3 fell after the peak of the stress–strain was reached, *H*_c_ exhibited a small “sudden rise,” and rebound deformation occurred in limestone specimens at a rate of 0.036% and an amplitude of 0.018 mm. Figure 12 shows that sudden drops in *H*_c_ occur when combined specimen B-2 falls after their respective stress–strain curve peaks; this is accompanied by rebound deformation in limestone specimens with an amplitude of 0.024 mm and a rate of 0.040%.

When the rock–coal inclination angle was less than or equal to 15°, it had an impact on the combined limestone specimens’ rebound deformation. As coal–rock inclination angles increased, rebound deformation’s amount and rate increased as observed in combined limestone specimens. However, when inclination angles exceeded or became equal to 30°, slip failure became predominant in combined specimens. It is worth noting that they did not exhibit any breakage or rebound deformation.

#### Exploring the mesoscopic properties of limestone

**Figure 13:**
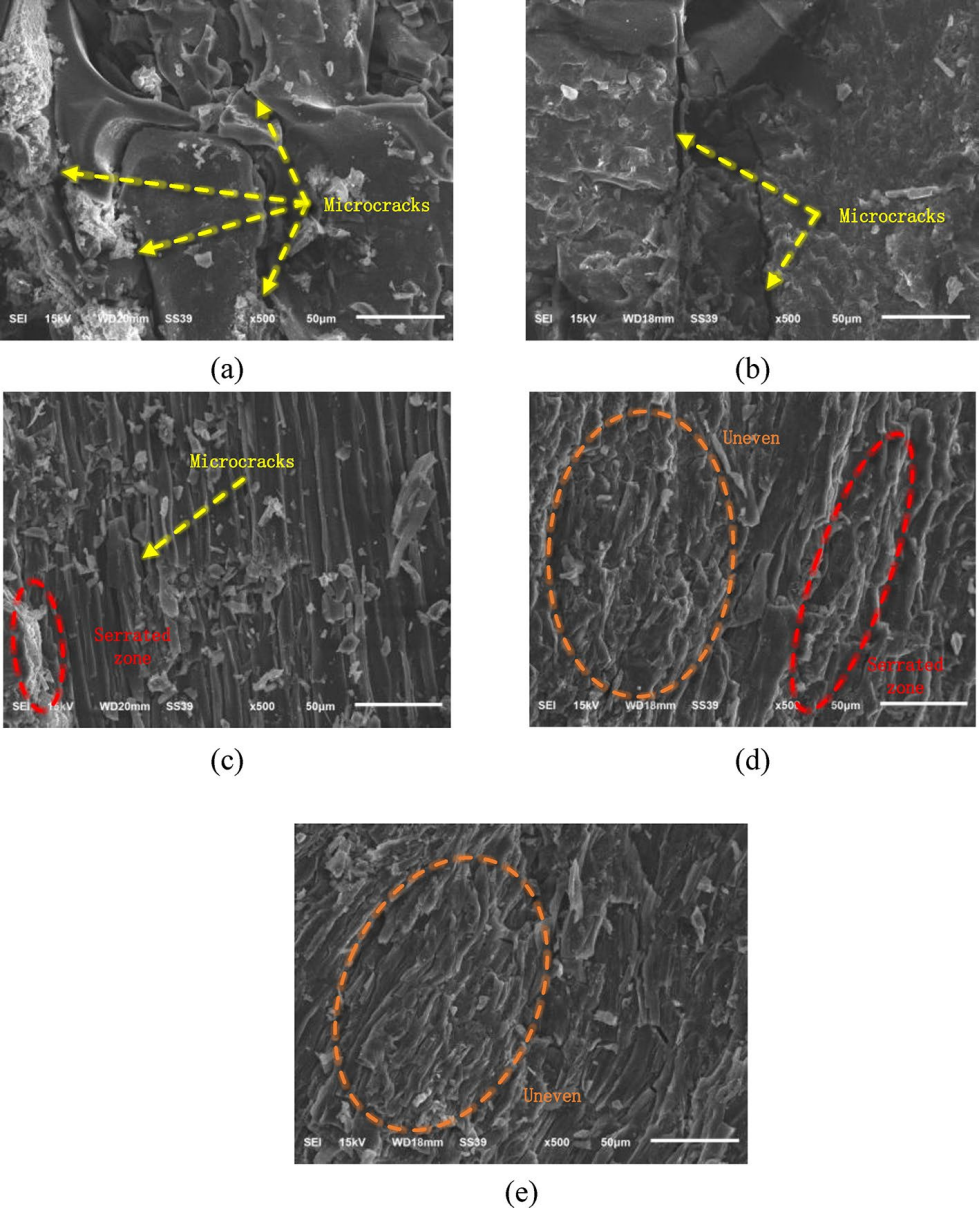
Scanning electron microscopy images of the fracture surfaces of combined limestone–coal specimens at different dip angles. Combined specimens (**a**) A-1, (**b**) B-1, (**c**) C-1, (**d**) D-1, and (**e**) E-1.

Refracture morphology can analyze the microscopic properties of the fracture surfaces of combined materials. Fracture surfaces at varying inclination angles contain abundant information; this information is highly significant for the examination of materials’ deformation and failure patterns from a microscopic perspective. The micro-properties of fractured coal specimen surfaces are shaped by the interplay among axial stress, inclination angle at the interface, and inherent flaws within coal specimens. These factors are closely associated with both rock–coal inclination angles. To conduct scanning electron microscopic imaging, we selected the fractured surfaces of combined specimens A-1, B-1, C-1, D-1, and E-1, as depicted in Fig. 13.

From Fig. 13, it is evident that decreased rock–coal inclination angles allow ample time for the development of microcracks on the fracture surface of the combined specimen. This facilitates the formation of a macroscopic fracture surface characterized by relative flatness and smoothness with underdeveloped microcracks. Furthermore, one can observe serrated areas on fractured surfaces where bite frictions from serrated steps partially suppress the failure of coal specimens. There is a noticeable “step-like” drop in the stress curve at the post-peak loading stage, thus indicating certain relative ductility in the failure of coal specimens. Conversely, an increase in inclination angle results in uneven fracture surfaces for coal specimens. This suggests that severe combined failure occurs without allowing sufficient time for developing and expanding fractured surfaces.

### Energy evolution and AE characteristics of limestone–coal composites

#### Analysis of energy evolution characteristics

The deformation and failure of rock coal assemblages are the result of energy-driven processes. According to the first law of thermodynamics, it is assumed that when an external force does work, there is no heat exchange between the combination and the outside world. In this experiment, the energy values of each part during the loading process of the combination can be calculated using formulas (3)–(8)^10,35^.

The input energy density $U_{{\text{I}}}$ generated by the external force is the sum of the elastic energy density $U_{{\text{E}}}$ and the dissipated energy density $U_{{\text{D}}}$.

3 $$U_{{\text{I}}} = U_{{\text{E}}} + U_{{\text{D}}}$$

**Figure 14:**
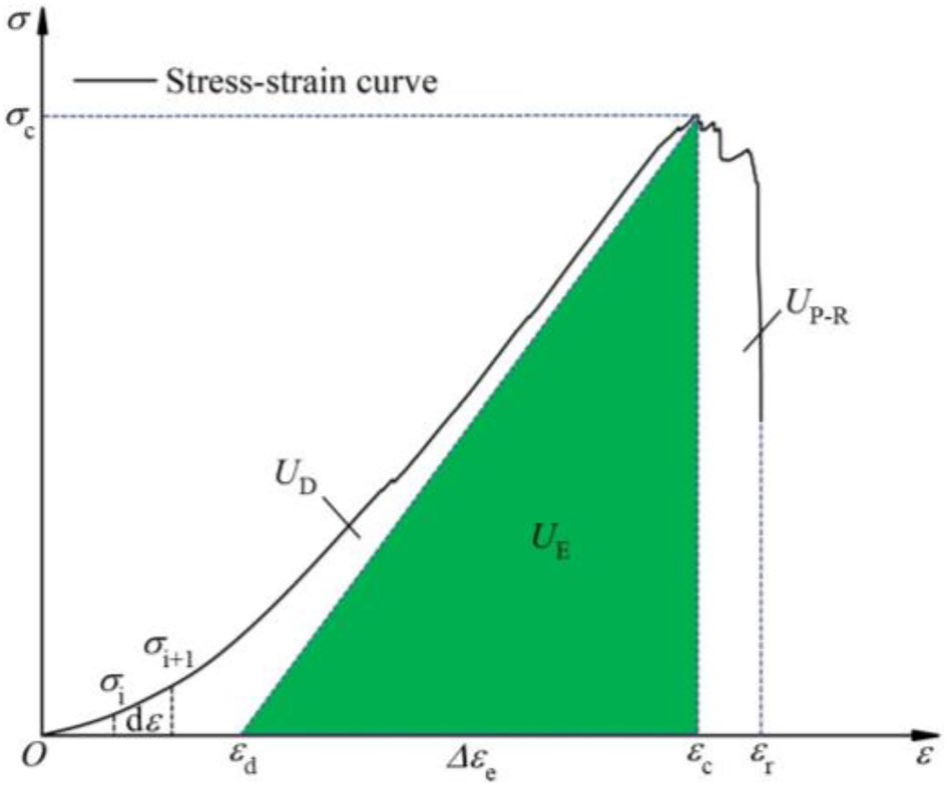
Energy density conversion relationship of combined limestone–coal specimens^36–38^.

Figure 14 shows the energy density conversion relationship of combined limestone–coal specimens. The calculation formula for input energy density $U_{{\text{I}}}$ can be obtained from Fig. 14.

4 $$U_{{\text{I}}} = \int_{0}^{{\varepsilon_{{\text{c}}} }} {\sigma_{{\text{i}}} } {\text{d}}\varepsilon$$

In the formula, $\sigma_{{\text{i}}}$ is the stress at any point on the stress–strain curve, MPa, and $\sigma_{{\text{c}}}$ is the strain corresponding to the peak stress.

The dissipation energy density $U_{{\text{D}}}$ and internal storage elastic energy density $U_{{\text{E}}}$ of the composite under load can be obtained from formulas (5) and (6), respectively.

5 $$U_{{\text{E}}} = \frac{1}{2}\sigma_{{\text{c}}} \left( {\varepsilon_{{\text{c}}} - \varepsilon_{{\text{d}}} } \right){ = }\frac{1}{2}\sigma_{{\text{c}}} \Delta \varepsilon_{{\text{e}}}$$

6 $$U_{{\text{D}}} = U_{{\text{I}}} - U_{{\text{E}}} = \int_{0}^{{\varepsilon_{{\text{c}}} }} {\sigma_{{\text{i}}} } {\text{d}}\varepsilon - \frac{1}{2}\sigma_{{\text{c}}} \Delta \varepsilon_{{\text{e}}}$$

In the formula, $\sigma_{{\text{c}}}$ is the peak stress, MPa, and $\left( {\varepsilon_{{\text{c}}} - \varepsilon_{{\text{d}}} } \right)$ represents recoverable strain.

The post-peak release energy density $U_{{\text{P - R}}}$ is the area of the stress–strain curve from the $\varepsilon_{{\text{c}}}$ to $\varepsilon_{{\text{r}}}$ envelope line, and its calculation formula is,

7 $$U_{{\text{P - R}}} = \int_{{\varepsilon_{{\text{r}}} }}^{{\varepsilon_{{\text{c}}} }} {\sigma_{{\text{i}}} } {\text{d}}\varepsilon$$

In the formula, $\varepsilon_{{\text{r}}}$ is the maximum strain of the stress–strain curve.

The calculation formula for the remaining elastic energy density $U_{{\text{R - E}}}$ is,

8 $$U_{{\text{R - E}}} = U_{{\text{E}}} - U_{{\text{P - R}}} = \frac{1}{2}\sigma_{{\text{c}}} \Delta \varepsilon_{{\text{e}}} - \int_{{\varepsilon_{{\text{r}}} }}^{{\varepsilon_{{\text{c}}} }} {\sigma_{{\text{i}}} } {\text{d}}\varepsilon$$

**Figure 15:**
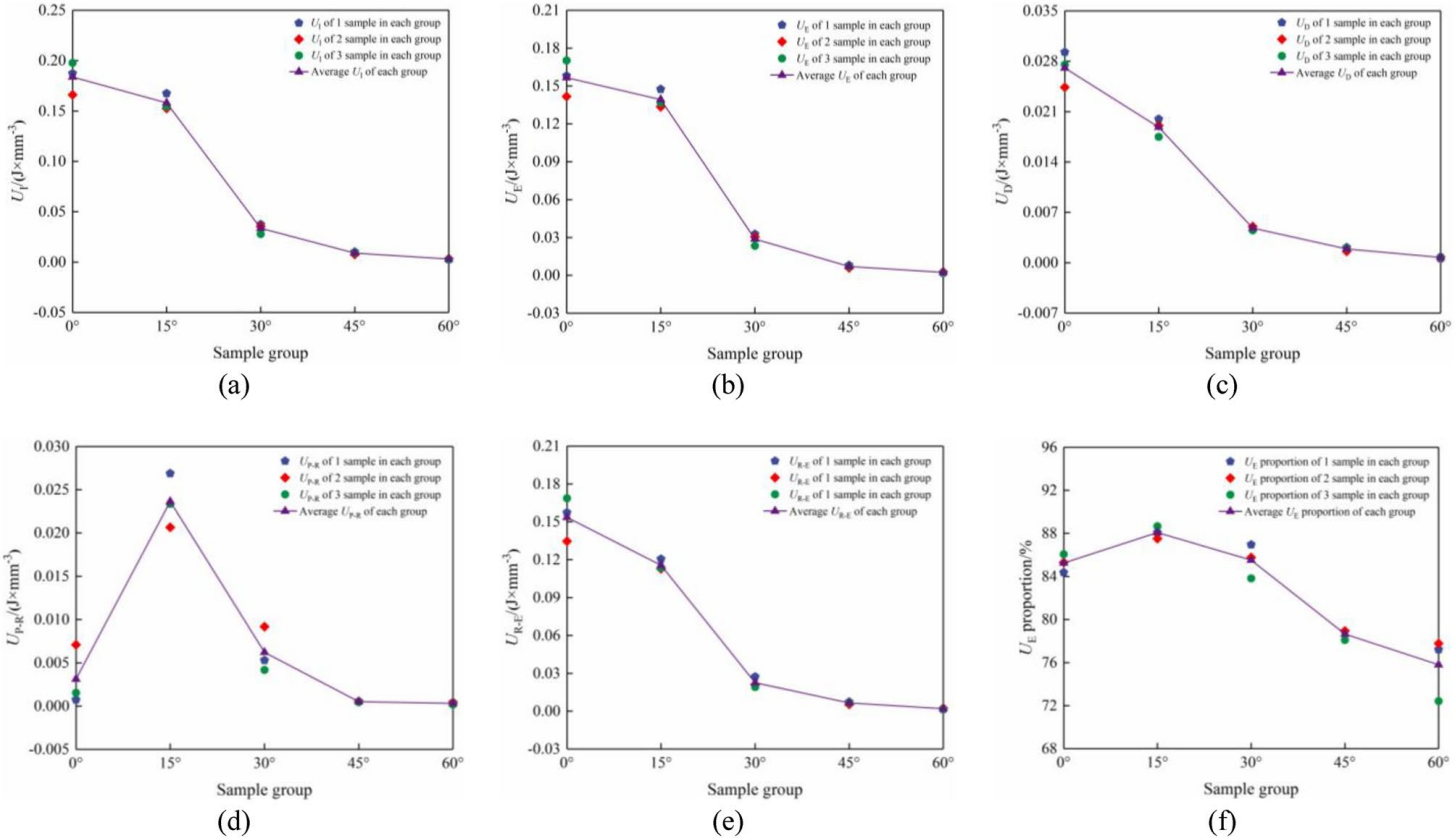
Comparison of proportions $U_{{\text{I}}}$, $U_{{\text{E}}}$, $U_{{\text{D}}}$, $U_{{\text{P - R}}}$, $U_{{\text{R - E}}}$, and $U_{{\text{E}}}$ of combined limestone–coal specimens at different dip angles. (**a**) $U_{{\text{I}}}$, (**b**) $U_{{\text{E}}}$, (**c**) $U_{{\text{D}}}$, (**d**) $U_{{\text{P - R}}}$, (**e**) $U_{{\text{R - E}}}$, and (**f**) $U_{{\text{E}}}$ proportion comparisons.

Figure 15 illustrates a comparison of energy densities observed in this experiment. As depicted in Fig. 15, as axial stress increases, there is a continuous external energy input. Combined specimens in group A exhibited the highest mean $U_{{\text{I}}}$ value (0.183741 J × mm^−3^), while combined specimens in group E had the lowest mean $U_{{\text{I}}}$ value (0.003056 J × mm^−3^). The failure of the combined specimens occurs only when the input energy reaches its storage limit. Consequently, it can be concluded that the strength of the combined specimens in group A was superior, thereby validating the accuracy of our test results. Additionally, among all groups, group A had the maximum mean $U_{{\text{E}}}$ value (0.156671 J × mm^−3^), whereas group E had the minimum mean value $U_{{\text{E}}}$ (0.002319 J × mm^−3^). It should be noted that any energy converted into an elastic property was stored within limestone and coal specimens.

The partial external energy was changed to dissipated energy associated with the failure of the combined specimens and internal deformation at the initial stage before reaching its peaks. Combined specimens in group A exhibited the highest mean value $U_{{\text{D}}}$ (0.003119 J × mm^−3^), while those in group E had the lowest mean value $U_{{\text{D}}}$ (0.000737 J × mm^−3^). As coal–rock inclination angles increased, dissipated energy transformation reduced during the pre-peak stage of the combined specimens; this resulted in less severe damage to the combined specimens and reduced plastic deformation before reaching peak stress. Combined specimens in group B had the largest mean proportion $U_{{\text{E}}}$ (88.08%), whereas those in group E had the smallest mean proportion $U_{{\text{E}}}$ (75.80%). With a rising coal–rock inclination angle, the combined specimens’ mean $U_{{\text{E}}}$ proportion first increased and was then decreased, thus indicating that the inclination angle at the interface influenced its ability to convert external energy into elastic properties. However, when rock and coal had an inclination angle ≤ 15°, the inclination angle at the interface did not have a significant effect. Inclination angles between coal and rocks reduced plastic deformation in combined specimens. Consequently, dissipated energy conversion during the pre-peak stage decreased while the $U_{{\text{E}}}$ proportion increased. When the inclination angles between coal and rocks exceeded or equaled 30°, this led to slip failure at the interface where separation occurred. Additionally, it caused storage capacity loss for $U_{{\text{E}}}$ and a subsequent reduction in the $U_{{\text{E}}}$ proportion within combined specimens.

Elastic-energy conversion generates residual elastic performance and releases energy at the post-peak stage. As the coal–rock inclination angles increased, the combined specimens’ mean value $U_{{\text{P - R}}}$ first increased and then decreased. Amongst the combined specimens in group B, the mean value $U_{{\text{P - R}}}$ was found to be the highest (0.023640 J × mm−^−3^), while those in group E yielded the lowest mean $U_{{\text{P - R}}}$ value (0.000323 J × mm^−3^). The maximum mean $U_{{\text{R - E}}}$ value was observed in the combined specimens in group A (0.153552 J × mm^−3^), whereas those in group E yielded the minimum mean $U_{{\text{R - E}}}$ value (0.001996 J × mm^−3^). For inclination angles ≤ 15°, the increased angle led to an escalation in macroscopic cracks within combined specimens; however, it resulted in a reduction in dynamic behavioral intensity. Conversely, when the inclination angles were ≥ 30°, the angle increases reduced the severity regarding the separation at the interface between coal and rocks within the combined specimens; these outcomes agreed with corresponding test outcomes.

### Analysis of AE characteristics

**Figure 16:**
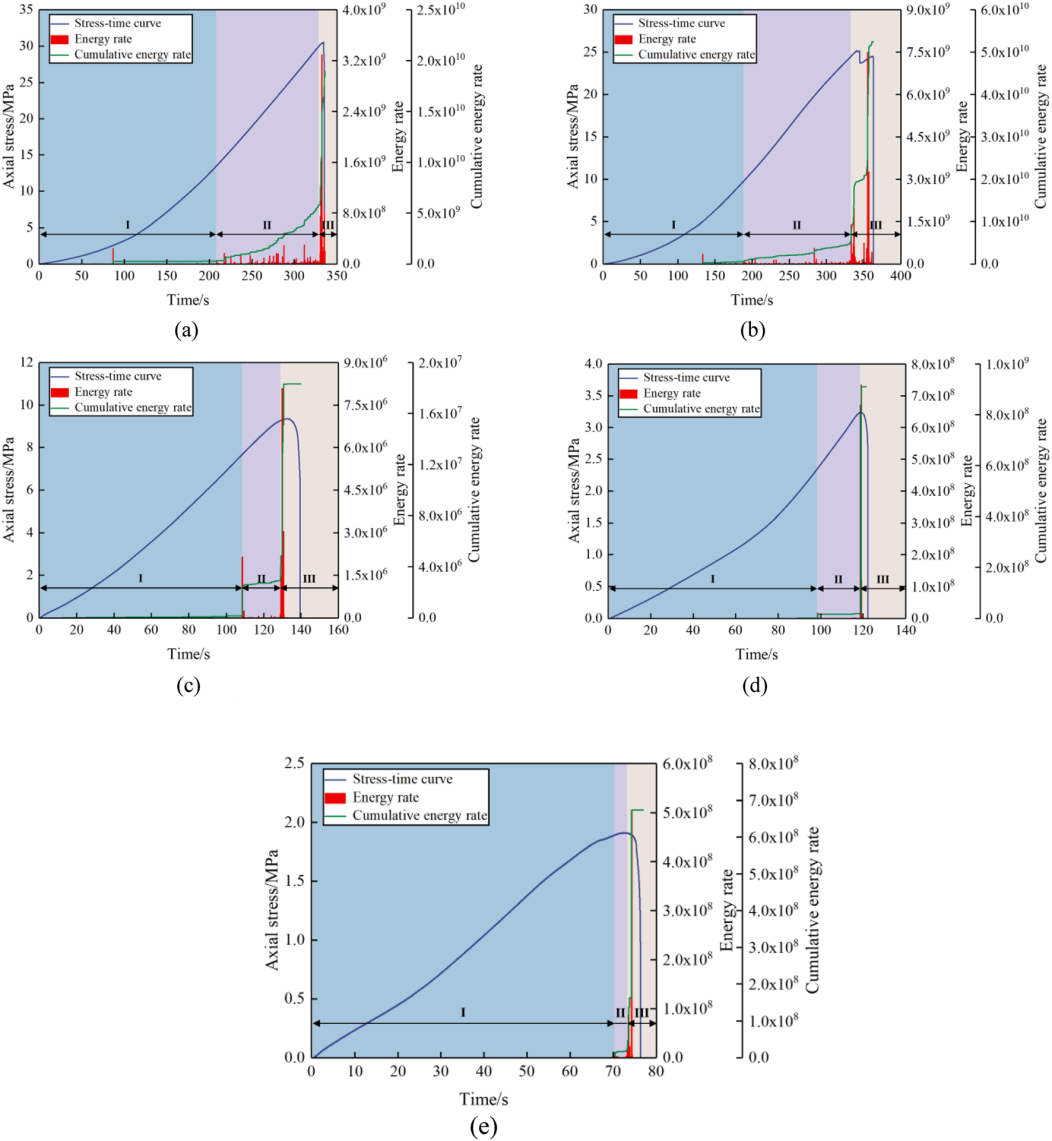
Characteristics of uniaxial compressive stress, acoustic emission energy rate, and cumulative energy rate of combined limestone–coal specimens at different dip angles. (**a**) A-1, (**b**) B-1, (**c**) C-1, (**d**) D-2, and (**e**) E-2.

The failure mechanism of the combined limestone–coal specimens involves continuous energy release to surrounding environments. AE technology can be employed to monitor elastic waves emitted by combined specimens, which are then converted into AE signals used for the characterization of their failure process. Furthermore, owing to variations in inclination angles, there are corresponding changes in AE signals and mechanical properties of the combined specimens. Hence, we selected the combined specimens A-1, B-1, C-1, D-2, and E-2 for the analysis of their cumulative energy rates and AE patterns during failure. Figure 16 illustrates characteristic curves depicting uniaxial compression stress along with cumulative and AE energy rates for each specimen.

As depicted in Fig. 16, AE energy rate signals displayed distinct stage properties as axial stress increased. By analyzing the quantities and peak values of these signals, we categorized the combined specimens’ progressive failure process under uniaxial compression into silent (stage I), active (stage II), and sudden escalation (stage III). Furthermore, a notable consistency exists between stress–time curves and AE energy rate signals throughout the combined specimens’ loading procedure.

1. Silent stage (Stage I)
  There was observed compaction and primary-defect closure at the contact interface between limestone and coal specimens subjected to uniaxial loading at the initial stage. Combined specimens exhibited linear elastic deformation, with most input energy stored as elastic energy within specimens. During this stage, no evident AE activities were detected in the combined specimens’ entire interior. Notably, when the inclination angle exceeded or was equal to 30°, both cumulative and AE energy rates were negligible. Combined specimens’ stage I showed an increase in cumulative energy rate accompanied by AE energy rate signals at the inclination angles of 0° and 15°. This can be attributed to a lesser influence from the interfacial dip angle effect on combined specimens owing to smaller inclination angles, thus leading to a slight enhancement in the AE energy rate and primary defects’ potential closure.
2. Active stage (Stage II)
  There was a noticeable change in the damaging quality and failure observed in the combined specimen during the active phase. Specimens A-1 and B-1 exhibited visible internal crack signs that gradually developed and indicated significant damage. Similarly, rock–coal interfaces in specimens C-1, D-2, and E-2 exhibited slipping signs. Moreover, there were notable fluctuations in AE energy rate signals in conjunction with a substantial rise in cumulative energy rate. As inclination angles increased, specimens A-1, B-1, C-1, D-2, and E-2 entered stage II at 207 s, 188 s, 108 s, 98 s, and 70 s respectively. It is worth noting that as inclination angles increased, combined specimens entering stage II decreased owing to an amplified tendency for slip along the interfaces between rocks and coal that resulted from the increases of the angle. This ultimately led to the failure of the combined specimens.
3. Sudden increase (Stage III)
  Combined specimens converted their stored elasticity energies into residual elastic properties and post-peak release energy during sudden changes. During this process, there was an abrupt increase in the AE energy rate signals; in this case, the cumulative energy rates reached their maximum values. Ultimately, when experiencing integral failure, combined specimens A-1, B-1, C-1, D-2, and E-2 exhibited the AE energy rates of 3.29 × 10^9^, 7.48 × 10^9^, 8.08 × 10^6^, 7.34 × 10^8^, and 5.05 × 10^8^, respectively; cumulative energy rates were measured at 1.89 × 10^10^, 5.25 × 10^10^, 1.83 × 10^7^, 9.11 × 10^8^, and 6.74 × 10^8^, respectively. When the inclination angles of combined specimens were ≥ 30°, significantly decreased levels of both energy and cumulative energy rates were demonstrated compared with those for inclination angles ≤ 15°. This can be attributed to increasing inclination angles in rocks and coal that led to interface slip that dominated the combined specimens’ failure mechanism. As a result, the combined specimens’ strength was greatly diminished along with its ability to store explosive potential. In other words, released energy and residual energy of elasticity after reaching peak transformation were minimal; this resulted in smaller macrocracks and decreased dynamic strength following failure.

### Informed consent

Informed consent was obtained from all subjects involved in the study.

## Conclusions

1. Each specimen group’s stress–strain curves showed plastic yield characteristics during the pre-peak stage. However, slight variations were observed due to the inclination angle’s effects. When the inclination angle was less than or equal to 15°, a minor “stress drop” phenomenon occurred at the plastic yield stage. When the inclination angles are greater than or equal to 30°, a tendency toward smoother responses occurred at increased inclination angles at the plastic yield stage. Furthermore, increasing inclination angles resulted in decreasing trends for both the uniaxial compressive strength and elastic modulus of combined specimens. Notably, between 15° and 30°, there was an instant rise in decreased amplitude due to interfacial inclination angle effects.
2. Localization zones for deformation evolution closely correlated with primary crack initiation and propagation in specimens as well as slip at interfaces between coal and rocks. For inclination angles less than or equal to 15°, coal specimens’ failure induced rebound deformation in limestone. As inclination angles increased, rebound deformation in limestone ranged from 0.018 to 0.024 mm while it exhibited an increasing trend for both rebound deformation amount and rate (from 0.036 to 0.040%). Conversely, when inclination angles were greater than or equal to 30°, the interfacial slip played a dominant role in the cases of combined specimens which promoted noncooperative deformation between coal and limestone at those interfaces.
3. Pre-peak elastic property density’s percentage in combined specimens increased from 98.56% to 88.08% at increasing inclination angles and was reduced to 75.80%. External energy was converted into missile performance, thus indicating an initial rise followed by a reduction. Similarly, the transformation of post-peak elastic energy into release energy initially increased and was then reduced, while transformations into the residual elastic property were reduced. Additionally, slip-failure severity was reduced at the rock–coal interface during failure.
4. The rate signal of AE energy exhibited distinct temporal characteristics in combined specimens; signal increases were classified into quiet, active, and sudden increases. When complete damage occurred in combined specimens, there was a significant reduction in energy and cumulative energy rates for specimens at inclination angles ≥ 30° compared with those at inclination angles ≤ 15°.

## Data Availability

The datasets used and/or analyzed during the current study are available from the corresponding author upon reasonable request.

## References

[1] Xie HP, Gao F, Ju Y (2015). Research and development of rock mechanics in deep ground engineering. Chin. J. Rock Mech. Eng..

[2] Petr K, Kamil S, Lubomir S (2013). Long-hole destress blasting for rockburst control during deep underground coal mining. Int. J. Rock Mech. Min. Sci..

[3] Kabiesz J, Lurka A, Drzewiecki J (2015). Selected methods of rock structure disintegration to control mining hazards. Arch. Min. Sci..

[4] Wierzbicki M, Młynarczuk M (2013). Structural aspects of gas and dolomite outburst in Rudna copper mine, Poland. Int. J. Rock Mech. Min. Sci..

[5] Fan YF, Xiao XC, Xu J (2023). Failure characteristics and conditions of rock-coal combination structure with weak layer under dynamic and static stresses. Sci. Rep..

[6] Lama RD, Bodziony J (1998). Management of outburst in underground coal mines. Int. J. Coal Geol..

[7] Chen SJ, Yin DW, Liu HM (2019). Effects of coal’s initial macro-cracks on rockburst tendency of rock-coal composite samples. R. Soc. Open Sci..

[8] Chen SJ, Yin DW, Jiang N (2019). Mechanical properties of oil shale-coal composite samples. Int. J. Rock Mech. Min. Sci..

[9] Yin DW, Chen SJ, Sun XZ (2019). Strength characteristics of roof rock-coal bi-material samples with different height ratios under uniaxial loading. Arch. Min. Sci..

[10] Yin DW, Chen SJ, Ge Y (2021). Mechanical properties of rock-coal bi-material samples with different lithologies under uniaxial loading. J. Market. Res..

[11] Chai YJ, Dou LM, Cai W (2023). Experimental investigation into damage and failure process of coal-rock composite structures with different roof lithologies under mining-induced stress loading. Int. J. Rock Mech. Min. Sci..

[12] Zhao ZH, Wang WM, Wang LH (2015). Compression-shear strength criterion of coal-rock combination model considering interface effect. Tunn. Undergr. Space Technol..

[13] Liu XS, Tan YL, Ning JG (2018). Mechanical properties and damage constitutive model of coal in coal-rock combined body. Int. J. Rock Mech. Min. Sci..

[14] Zuo JP, Wang ZF, Zhou HW (2013). Failure behavior of a rock-coal-rock combined body with a weak coal interlayer. Int. J. Min. Sci. Technol..

[15] Liu J, Wang EY, Song DZ (2015). Effect of rock strength on failure mode and mechanical behavior of composite samples. Arab. J. Geosci..

[16] Huang BX, Liu JW (2013). The effect of loading rate on the behavior of samples composed of coal and rock. Int. J. Rock Mech. Min. Sci..

[17] Yin DW (2018). Effect of joint angle in coal on failure mechanical behavior of rock-coal combined body. Q. J. Eng. Geol. Hydrogeol..

[18] Yin DW, Meng XX (2019). Numerical simulation on uniaxial compression Failure of a roof rock-coal-floor rock composite sample with coal persistent joint. Geotech. Geol. Eng..

[19] Yin DW, Chen SJ, Chen B (2018). Strength and failure characteristics of the rock-coal combined body with single joint in coal. Geomech. Eng..

[20] Li YK, Liu JW, Yu Q (2022). Patterns of influence of parallel rock fractures on the mechanical properties of the rock–coal combined body. Sustainability.

[21] Guo WY, Tan YL, Yu FH (2018). Mechanical behavior of rock-coal-rock specimens with different coal thicknesses. Geomech. Eng..

[22] Zhao TB, Guo WY, Lu CP (2016). Failure characteristics of combined coal-rock with different interfacial angles. Geomech. Eng..

[23] Nie X, Zhou AC (2018). Numerical analysis on mechanical characteristics of coal-rock combination of different height ratios. Coal Technol..

[24] Zhao HL, Zhao Y (2018). Influence of dip angle of coal and rock combination on outburst tendency based on particle flow code. Saf. Coal Mines.

[25] Zhao ZH, Wang WM, Dai CQ (2014). Failure characteristics of three-body model composed of rock and coal with different strength and stiffness. Trans. Nonferrous Met. Soc. China.

[26] Li FX, Yin DW, Zhu C (2021). Effects of Kaolin addition on mechanical properties for cemented coal gangue-fly ash backfill under uniaxial loading. Energies.

[27] Huang DM, Zhu YY, Qiao SY (2023). Numerical experiments on macro-and micro-damage characteristics of red sandstone with pore-double fractures. J. Shandong Univ. Sci. Technol. (Natural Science).

[28] Wan GQ, Huang F, Liu XC (2020). Application of digital speckle technique in concrete uniaxial compression test. Opt. Tech..

[29] Ma XY, Gong XF, Bian LG (2022). Basic mechanical properties and failure mechanism of red sandstoneunder biaxial loading condition. J. Shandong Univ. Sci. Technol. (Natural Science).

[30] Li YY, Yan HD, Zhang SC (2022). Experimental study on the expansion law and mechanical characteristics of crack propagation in rock with composite defect. J. Shandong Univ. Sci. Technol. (Natural Science).

[31] Yu WJ, Wu GS, Liu Z (2020). Uniaxial compression test of coal-rock-bolt anchorage body and mechanical mechanisms of bolts. Chin. J. Rock Mech. Eng..

[32] Zheng J, Yao XX, Chen Y (1983). Experimental study on localization of rock deformation. Acta Geophys. Sin..

[33] Song YM, Jiang YD, Ma SP (2012). Evolution of deformation fields and energy in whole process of rock failure. Rock Soil Mech..

[34] Mikhalyuk AV, Zakharov VV (1997). Dissipation of dynamic-loading energy in quasi-elastic deformation processes in rocks. J. Appl. Mech. Tech. Phys..

[35] Xie HP, Li LY, Ju Y (2011). Energy analysis for damage and catastrophic failure of rocks. Sci. China Technol. Sci..

[36] Jansen DC, Shah SP (1997). Effect of length on compressive strain softening of concrete. J. Eng. Mech..

[37] Accornero F, Cafarelli R, Carpinteri A (2022). The cohesive/overlapping crack model for plain and RC beams: Scale effects on cracking and crushing failures. Mag. Concr. Res..

[38] Carpinteri A, Lacidogna G, Corrado M (2016). Cracking and crackling in concrete-like materials: A dynamic energy balance. Eng. Fract. Mech..

